# Secondary Structure Analyses of the Nuclear rRNA Internal Transcribed Spacers and Assessment of Its Phylogenetic Utility across the Brassicaceae (Mustards)

**DOI:** 10.1371/journal.pone.0101341

**Published:** 2014-07-01

**Authors:** Patrick P. Edger, Michelle Tang, Kevin A. Bird, Dustin R. Mayfield, Gavin Conant, Klaus Mummenhoff, Marcus A. Koch, J. Chris Pires

**Affiliations:** 1 Division of Biological Sciences, University of Missouri, Columbia, Missouri, United States of America; 2 Department of Plant and Microbial Biology, University of California Berkeley, Berkeley, California, United States of America; 3 Informatics Institute, University of Missouri, Columbia, Missouri, United States of America; 4 Division of Animal Sciences, University of Missouri, Columbia, Missouri, United States of America; 5 Department of Biology, University of Osnabrück, Osnabrück, Germany; 6 Department of Biodiversity and Plant Systematics, Heidelberg University, Heidelberg, Germany; University of Gottingen, Germany

## Abstract

The internal transcribed spacers of the nuclear ribosomal RNA gene cluster, termed ITS1 and ITS2, are the most frequently used nuclear markers for phylogenetic analyses across many eukaryotic groups including most plant families. The reasons for the popularity of these markers include: 1.) Ease of amplification due to high copy number of the gene clusters, 2.) Available cost-effective methods and highly conserved primers, 3.) Rapidly evolving markers (i.e. variable between closely related species), and 4.) The assumption (and/or treatment) that these sequences are non-functional, neutrally evolving phylogenetic markers. Here, our analyses of ITS1 and ITS2 for 50 species suggest that both sequences are instead under selective constraints to preserve proper secondary structure, likely to maintain complete self-splicing functions, and thus are not neutrally-evolving phylogenetic markers. Our results indicate the majority of sequence sites are co-evolving with other positions to form proper secondary structure, which has implications for phylogenetic inference. We also found that the lowest energy state and total number of possible alternate secondary structures are highly significantly different between ITS regions and random sequences with an identical overall length and Guanine-Cytosine (GC) content. Lastly, we review recent evidence highlighting some additional problematic issues with using these regions as the sole markers for phylogenetic studies, and thus strongly recommend additional markers and cost-effective approaches for future studies to estimate phylogenetic relationships.

## Introduction

**Figure 1:**
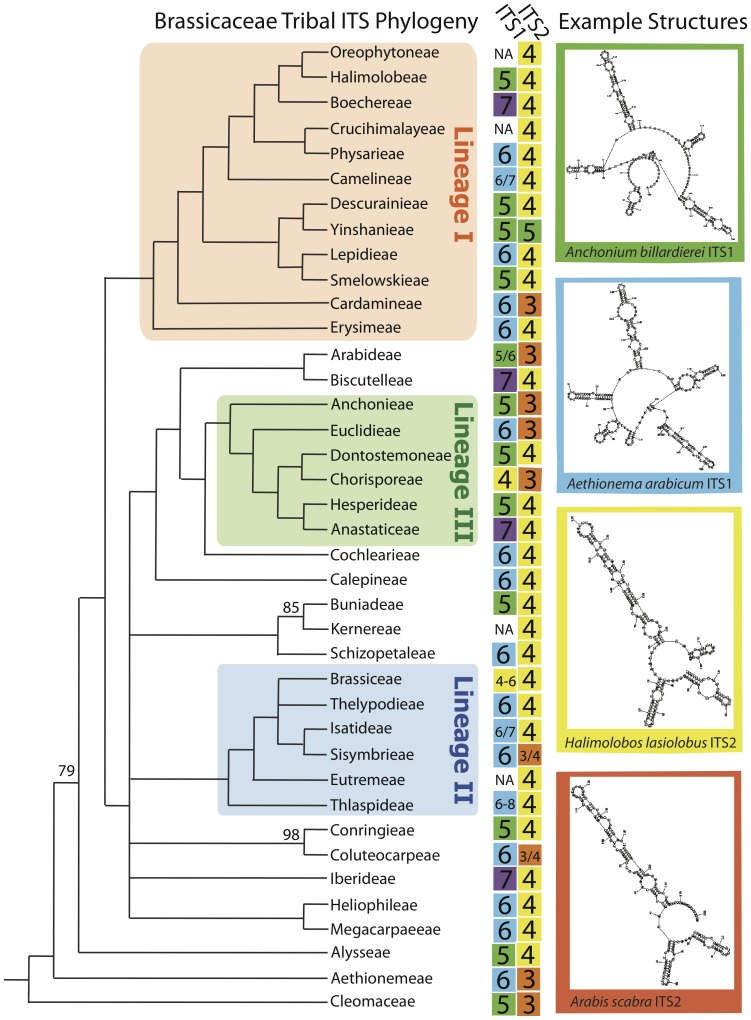
Phylogenetic Distribution of Hairpin Numbers for ITS Secondary Structures. A tribal level phylogeny of the Brassicaceae, strict consensus tree of the 200 most parsimonious trees estimated with ITS sequences [2], was utilized to investigate the evolution of the number of hairpins present in the secondary structures of both ITS1 and ITS2. Bootstrap support values greater than 60% are shown above branches [2]. It is notable that the ITS tree does neither fully reflect the tribal phylogeny nor is at any deep node highly significantly supported, but overall-topology is in congruence with multi-locus phylogenies considering major lineages [7], [8]. Tribes not assigned to one of the three major lineages are actually combined with an "expanded lineage II" [46], which might have to be revised in future. The three major phylogenetic lineages are shown within colored blocks with Lineage I (orange), Lineage II (blue) and Lineage III (green). The number of hairpins for each secondary structure is shown at the phylogenetic tips with 3 (orange boxes), 4 (yellow boxes), 5 (green boxes), 6 (blue boxes), and 7 (purple boxes). Tribes with a lack of available complete ITS1 data are marked as 'NA'. Tribes with secondary structures with different number of total hairpins from different species are also indicated (e.g. Camelineae 6/7 for ITS1; 6 and 7 hairpin structures are observed) within the colored box of the fewest hairpined structure. Examples of secondary structures are shown (top-bottom order): 1. *Anchonium billardierei* ITS1 (Anchonieae), 2. *Aethionema arabicum* ITS1 (Aethionemeae), 3. *Halimolobos lasiolaba* ITS2 (Halimolobeae), and 4. *Arabis scabra* (Arabideae).

Molecular systematic approaches have traditionally relied on comparing a limited number of orthologous sequences to obtain estimates of species relationships across the tree of life. These phylogenetic markers are often selected based on a number of basic characteristics; including 1.) Ubiquitous presence across target taxa, 2.) Sufficient sequence or structural variation between taxa (i.e. synapomorphic characters), 3.) Ease of obtaining sequence data, 4.) Cost-effectiveness, and 5.) Having a fundamental understanding of the function of the locus and the possible selective forces acting on its sequence evolution. The internal transcribed spacers (ITS1 and ITS2) regions of the ribosomal RNA gene cluster are the most commonly used nuclear markers for estimating species relationships across plants based on the above criteria, including the assumption (or treatment) that these markers are non-functional and hence neutrally evolving. For example, the ITS regions are the most commonly used markers for estimating phylogenetic relationships across the mustard family (Brassicaceae) (**Figure 1**) [1], [2], with sequences available for the majority of the 321 genera distributed across all 49 delimited tribes [3]. These studies have, in addition to delimiting tribes and estimating phylogenetic relationships among some tribes, also been successful at assigning many of the tribes into one of three monophyletic lineages (**Figure 1**). Lineage I is comprised of twelve tribes including Camelineae that contains the model organism *Arabidopsis thaliana.* Lineage II has six tribes including the agronomically important Brassiceae that contains cruciferous vegetables (e.g cabbage, cauliflower, broccoli, and brussel sprouts). Lineage III includes six primarily Asian tribes. It is important to note that other phylogenetic markers have also been employed (see discussion)[4]–[8]; nonetheless more than 70 articles published since 2010 have used the ITS region to infer relationships across the Brassicaceae.

**Figure 2:**
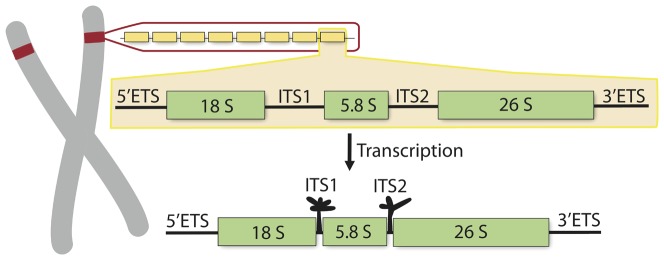
Structure of the rDNA region in Plants. An illustration of the nucleolus organizing region (NOR), shown as red colored region on chromosome, is associated with forming the nucleolus and site for the biosynthesis of the components of the ribosome. The NOR region contains hundreds of tandem duplicated copies of rDNA gene clusters (depicted as yellow rectangles), and each gene cluster consists of seven main components including two internal transcribed spacers (i.e. ITS1 and ITS2). These ITS regions form self-splicing secondary structures as transcribed products. Shown is the 5 hairpin structure for ITS1 and the 3 hairpin structure for ITS2.

The ribosomal RNA gene cluster consists of seven components: the 5′ external transcribed spacer, the 18S rDNA exon, internal transcribed spacer 1 (ITS1), the 5.8S rDNA exon, internal transcribed spacer 2 (ITS2), the 28S rDNA exon, and the 3′ external transcribed spacer (**Figure 2**) [9]. The rDNA exons are highly conserved across eukaryotes, but the ITS regions are variable in length due to point mutations and indels, resulting in regions varying in size from 500 to 700 bp across angiosperms [10] and from 1500–3700 bp in some gymnosperms [11]. The ITS regions are not incorporated into mature ribosomes, but undergo a specific cleavage during the maturation of the ribosomal RNAs that is catalysed by the secondary structure of ITS sequences themselves [12]–[15]. Despite this specific activity, these sequences have been treated as nearly neutrally evolving nuclear markers for phylogenetic reconstructions [16], for reviews see [17]–[19]. Here, we test whether nuclear encoded internal transcribed spacers (ITS1 and ITS2) are truly neutrally evolving, or if these regions are under selective constraints to maintain a functional self-splicing secondary structure across the Brassicaceae. In addition, we assess the phylogenetic utility of secondary structure data for inferring phylogenetic relationships.

## Methods

All possible ITS1 and ITS2 secondary structures for a total of 50 species (100 total structures) and 100 random sequences were modeled using RNAstructure Version 5.3 [20]. **Sheet 1 in File S1** contains the list of species, NCBI GenBank accession numbers, lengths, total number of possible secondary structures, and a description of the lowest energy state structure for every species. All ITS sequences have been verified by taxonomic experts [2], annotated and deposited into BrassiBase (a comprehensive Brassicaceae database system; http://brassibase.cos.uni-heidelberg.de/) and within a proven phylogenetic context [21], [22]. The 100 random sequences, with an overall GC content and size variation identical to these 100 ITS sequences (**Sheet 2 in File S1**) were generated using a custom Perl script. A two-way ANOVA, using the R software package (Version 1.7.1) [23], was used to test the statistical significance between both the lowest energy state and total number of possible structures between the true ITS sequences and random sequences (**Sheet 3 in File S1**). The structures were further analyzed manually for the total number of paired bases and number of total hairpins (**Sheet 4 & 5 in File S1**). Hairpins were characterized as a complete, continuous loop formed by a set of closely paired nucleotides between two distant regions, with either single or branched structures that may include additional nested structures, while stems were characterized as structures that do not form immediate loops. A structure required a minimum of four nucleotide bonds (i.e. eight nucleotides) to be characterized as a hairpin. Single and branched structures were both treated as one hairpin. Sequence alignments for ITS1 and ITS2 can be found in **Files S2** **& S3**, respectively.

## Results

**Figure 3:**
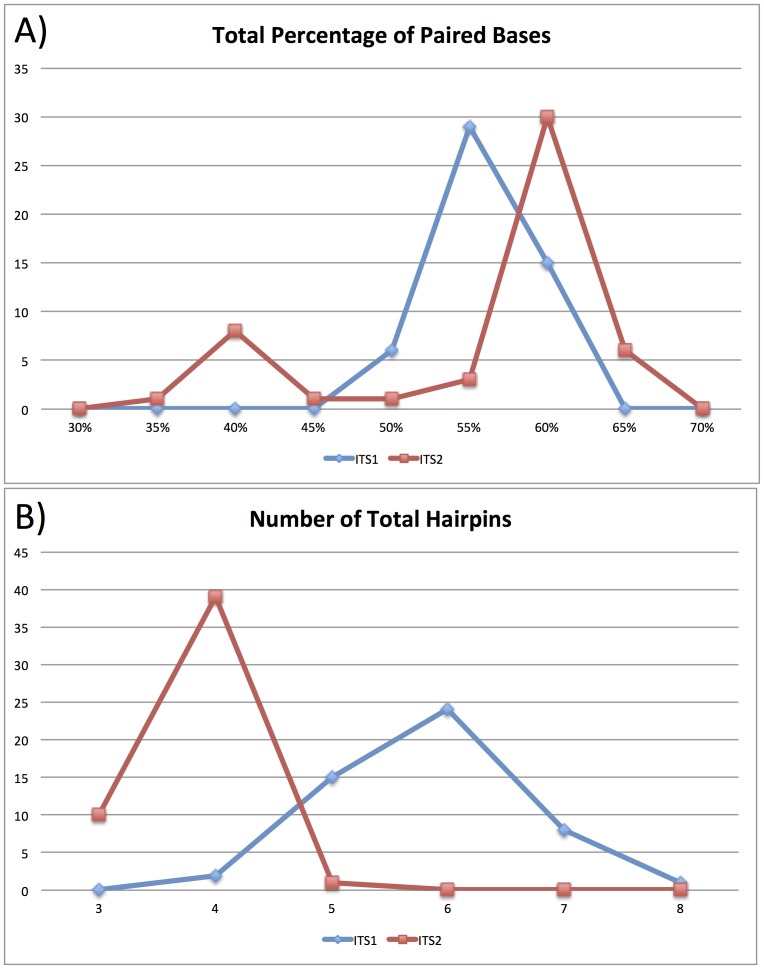
Hairpin Size Distributions for ITS1 and ITS2 Secondary Structures. Panel A shows the frequency of the total percentage of paired bases for all ITS1 (Blue) and ITS2 (Red) sequences. The majority of both ITS regions have over 50% of positions paired with other sequence positions to form secondary structures. Panel B shows the frequency of the total number of hairpins for all the ITS1 (Blue) and ITS2 (Red) structures. These data are for the lowest energy state structure for each ITS sequence (see Supplemental File 1).

All possible secondary structures for both ITS1 and ITS2 were modeled for 49 species distributed across 38 tribes in the family Brassicaceae and one outgroup species (*Cleome lutea* renamed *Peritoma lutea* Hook. [24]; Cleomaceae) (**Figure 3**). The ITS1 and ITS2 regions are variable in length. ITS1 has a mean length of 263bp (max = 286bp; min  = 238bp). ITS2 has a mean length of 184bp (max = 220bp; min = 177bp). The combined ITS1 and ITS2 sequences have a mean length of 224bp and a median length of 229bp. These ITS1 and ITS2 regions have a mean of 16.6 and 7.9 possible secondary structures, respectively. The combined ITS1 and ITS2 dataset has a mean of 10.5 possible secondary structures (median = 8.5; max = 30; min = 1). The mean for the lowest energy structure for all ITS1 and ITS2 sequences is −93.1 and −70.0 degrees, respectively. The combined ITS1 and ITS2 dataset has a mean of −81.6 degrees and median of −79.5 degrees for the lowest energy states (max = −58.2 degrees; min −114.5 degrees). The ITS1 structures have a mean of 153.8 paired bases (i.e. form bonds with other bases) (median = 154) and a mean of 5.8 total hairpins (median = 6). The ITS2 structures have a mean of 115 paired bases (median = 114) and a mean of 3.8 total hairpins (median = 4). The ITS1 sequences on average have ∼58.5% of all nucleotide positions paired, while ITS2 sequences on average have ∼62.5% of all nucleotide positions paired.

**Figure 4:**
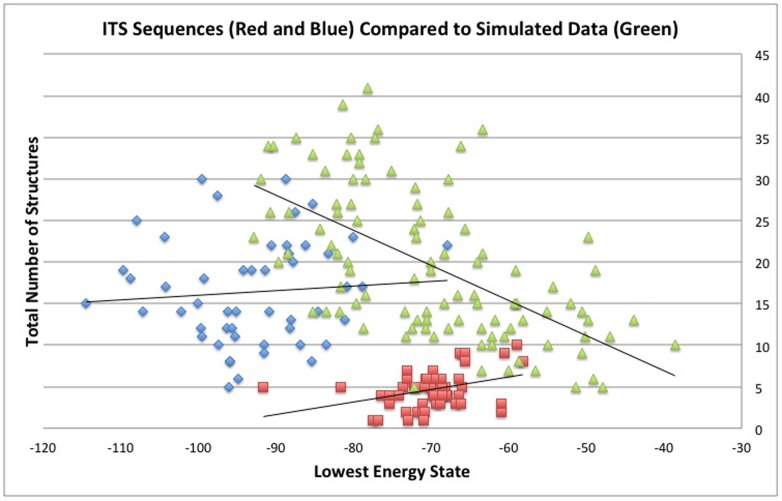
Comparison of Secondary Structures of 100 ITS and 100 Random Sequences. A scatter plot of the lowest energy state values (x-axis) and all possible secondary structures (y-axis) for 50 ITS1 (Blue Diamonds), 50 ITS2 (Red Squares) and 100 randomly generated sequences (Green Triangles) (Supplemental File 1) estimated using RNAstructure 5.3 (Reuter and Mathews, 2010).

To test if the secondary structures of the lowest energy states for ITS sequences suggest selective constraint on the sequences encoding them, we generated 100 random sequences with an identical size range (286bp to 177bp) and overall average GC content (54.9%; see **Sheet 2 in File S1)**. All possible secondary structures were modeled for each of the random sequences. These random sequences have a mean of 19.6 possible secondary structures (median = 17.5; max = 41; min = 5). The mean for the lowest energy structures for all random sequences is −69.9 (median = −70.7; max = −38.6; min = −92.8). We evaluated using a two-way ANOVA whether both the values of the lowest energy states and values for total possible secondary structures were significantly different between the 100 ITS and 100 random sequences (**Figure 4**). The lowest energy state and total number of possible secondary structures were both highly significant, (P<2.2e-16) and (P<1.6e-07), respectively. The ratio of both these categories was not significantly different between ITS and random sequences (P = 0.1095), as would be predicted. Similarly, the lowest energy state and total number of possible secondary structures for the 50 ITS1 sequences alone compared to random sequences were also significantly different, (P<2e-16) and (P<1.2e-04), respectively. The 50 ITS2 sequences compared to random sequences were also significantly different for both the the lowest energy state (P<2.1e-07) and total number of possible secondary structures (P<6.5e-08).

Lastly, we evaluated the utility of secondary structure data to resolve phylogenetic relationships at the tribal level across the Brassicaceae (**Figure 1**). ITS1 sequences are highly variable, ranging between 4 to 8 hairpins, with some conserved phylogenetic patterns within tribes but limited phylogenetic conservation between closely related tribes. Six hairpin structures for ITS1 are the most frequent for Lineage I (min = 5; max = 7) and Lineage II species (min = 4; max = 7), while 5 hairpin structures were most frequent among Lineage III species (Min = 4; Max = 7). ITS2 sequences are more slowly evolving, ranging between 3 to 5 hairpins, with most species having 4 hairpin secondary structures. Lineage I consists largely of 4 hairpin structures (min = 3; max = 5) and Lineage III consists of both 3 and 4 hairpin structures for ITS2. Lineage II species consist mostly of 4 hairpin structure ITS2 sequences, except for one species (*Sisymbrium irio*) with a 3 hairpin structure within the tribe Sisymbrieae. The ancestral state for the most common recent ancestor of the Brassicaceae is likely a 5 or 6 hairpin structure for ITS1, and a 3 or 4 hairpin structure for ITS2.

## Discussion

The results of this study show that the majority of sequence sites for both ITS1 and ITS2 are not independently evolving, but rather are co-evolving with at least one other position to preserve the molecule's secondary structure. Our analyses also revealed that these secondary structures have a significantly lower energy state and significantly fewer possible alternate secondary structures compared to random sequences with a similar guanine-cytosine (GC) content and length distribution. Collectively, these results do not support the neutrality of these sequences across the Brassicaceae (as commonly assumed and/or implemented for phylogenetic analyses), but rather strongly suggest that these sequences are under selective constraint to maintain functional self-splicing secondary structures. Thus, the majority of mutations that occur within these regions must likely undergo compensatory mutations, since most sites are co-evolving, to maintain properly functioning secondary structures.

Many Brassicaceae studies to date have used ITS as a phylogenetic marker to estimate relationships and delimit new taxonomic groups (e.g. tribes). Based on our results, this fact means that many phylogenetically informative sites were unintentionally treated as independent despite the fact that they were co-evolving. At a minimum, such assumptions will tend to overstate the confidence in phylogenetic hypotheses as inferred with methods such as the bootstrap. Thus, we advise future studies to identify all co-evolving sites and make appropriate adjustments prior to employing these regions as phylogenetic markers. However, we also want to emphasize the importance of proper consistent annotation prior to modeling secondary structures, particularly if structures are to be compared across species [25], [26]. For this reason, we generated random sequences with an identical length variation and overall GC content to actual ITS sequences to permit accurate comparisons of secondary structure characteristics. For the Brassicaceae community, the BrassiBase database has nearly 2,000 annotated ITS sequences and alignments are available for species distributed across all family tribes. For the broader eukaryotic community, the ITS2 Database [27] is an outstanding publicly available resource for the annotation, secondary structure prediction, and estimating phylogenetic relationships of ITS2 sequences.

For estimates of tribal relationships across the Brassicaceae (or likely for similar family level phylogenies), ITS1 and ITS2 contains insufficient signal to obtain a robust, well-resolved phylogeny (**Figure 1**). Thus, ITS must be combined with other markers to estimate deep level phylogenetic relationships. Additionally, the ITS marker is known to often undergo gene conversion following hybridization and allopolyploidization events, in which the sequence from one subgenome replaces those of the other subgenome [28]–[30]. For example, this homogenization process of rDNA repeats (i.e. concerted evolution) has been shown to occur very rapidly, within less than 100 years, in two allopolyploid *Cardamine* (Brassicaceae) species [31], and multiple populations of allopolyploid *Tragopogon mirus* and *Tragopogon miscellus* [32]. Also, the process of non-concerted evolution and the origin of paralogous copies have been described in the Brassicaceae [30]. Thus, these features and evolutionary histories of ITS sequences are not ideal for estimating species relationships alone, especially for groups like the Brassicaceae that have multiple documented ancient and recent polyploidization events [33]–[35]. Although reliance on ITS as the sole source of phylogenetic evidence can be criticized for reasons given here, it remains a highly efficient locus for generating species-level phylogenetic inferences in most plant groups. At least across the Brassicaceae, the phylogenetic estimates obtained from the ITS markers for within various subfamilial units have largely been congruent with other markers and data [36]–[38]. On the other hand, at the entire family level (i.e. tribes and major lineages), the phylogenetic signal from this marker has been insufficient to resolve major relationships.

Single-marker approaches are known to produce misleading phylogenetic estimates for species relationships [39], but incongruence between gene phylogenies and species phylogenies can be identified and resolved using multiple independently evolving markers [40]–[43]. More recent family-wide Brassicaceae studies are utilizing multi-locus datasets, which have yielded improved phylogenetic estimates for many clades but still limited resolution for the relationship among the three major lineages (shown in **Figure 1**) and majority of tribes [44]–[46]. Therefore future studies should survey additional markers with sufficient phylogenetic signal, preferably with different patterns of inheritance (e.g. mitochondrial, plastid, and nuclear), to estimate species relationships, identify incongruent markers with unique evolutionary histories, and ultimately obtain better insights into more complex evolutionary processes. A large-scale data approach has already been demonstrated to have sufficient signal to resolve some difficult phylogenetic relationships across the Brassicaceae [47], [48], and will serve as a valuable resource to address a range of fundamental questions in evolution remaining for the family including understanding the mechanisms responsible for shifts in speciation rates [49], evolution of chemical defenses against herbivores [50], and improve our understanding of novel morphological varation [51].

## Supporting Information

File S1 **Secondary Structure data for ITS1, ITS2, and random sequences.** Spreadsheet contains the list of species, NCBI GenBank accession numbers, lengths, total number of possible secondary structures, and a description of the lowest energy state structure for every ITS1, ITS2, and random sequence.
(XLSX)

File S2 **Sequence alignments for ITS1.** Sequence alignments for ITS1 for 49 Brassicaceae and one outgroup Cleomaceae found in File S1.
(TXT)

File S3 **Sequence alignments for ITS2.** Sequence alignments for ITS2 for 49 Brassicaceae and one outgroup Cleomaceae found in File S1.
(TXT)
